# Error Concealment in the Density Field of a Spatiotemporal Image Sequence

**DOI:** 10.1155/2022/4657431

**Published:** 2022-05-13

**Authors:** S. Rajarajeswari, C. Umarani, Anil Audumbar Pise, Dimitrios A. Karras, M. P. Karthikeyan, Sajjad Shaukat Jamal, Reynah Akwafo

**Affiliations:** ^1^M S Ramaiah Institute of Technology, Bengaluru 560054, Karnataka, India; ^2^Jain (deemed-to-be) University, Bengaluru, Karnataka, India; ^3^University of the Witwatersrand Johannesburg, South Africa; ^4^Department of Sustainable Engineering, Saveetha School of Engineering, Saveetha Institute of Medical and Technical Sciences, Saveetha University, Saveetha Nagar, Thandalam, Chennai, Tamil Nadu, India; ^5^National and Kapodistrian,University of Athens (NKUA), School of Science, Dept. General, Athens, Greece; ^6^School of CS and IT, Jain (Deemed-to-be University), Bengaluru, Karnataka, India; ^7^Department of Mathematics, College of Science, King Khalid University, Abha, Saudi Arabia; ^8^Electrical and Electronics Engineering Bolgatanga Technical University Ghana, Sumbrungu, Ghana

## Abstract

One of the most difficult challenges of multimedia transmission during the last two decades has been the retrieval of degraded or missing regions of images and videos while maintaining satisfactory perceptual accuracy. The objective is to retrieve lost data by using the similarity between frames. Usually, error concealment (EC) schemes depend on replacing incorrect data with data that are identical to the initial. This is possible because video contains a high degree of self-similarity. This research focuses on applying an EC approach in transform-domain video sequences. To conduct EC on films, they must first be translated to frames and then transformed using one of the available transformations into frequency-domain images. Using successive frames, it is possible to recover lost or incorrect data from images. Intra-coded frames (I-frames) may be used to recreate lost knowledge in predictive (P-frames) and bidirectional predictive frames (B-frames). I-frame knowledge that has been lost may be restored using previous intra-coded frames. The use of wavelet error concealment generated more precise results than the other techniques. In this study, it was discovered that covering faults in the density sector with wavelets produces more reliable results than the other techniques.

## 1. Introduction

Generally, the standard of service is not assured during online video transmission. If files have been made or corrupt, reinstalling them is a huge pain, but seeing them as bad as before and seeing them again as well is much worse. As the information is subjected to poor channel conditions, its transmission via the coded/decoding process is threatened, data may be corrupted, or it may have errors at the receiving end. Although avoiding packet losses or compromised data may often help mitigate quality loss due to transmitting errors; in general, data reduction (also known as error concealment) usually affects the overall quality of the video. When errors are concealed, one can discover the missing details by expanding the preceding information or unnecessary information [1]. Because transmitting errors happen throughout the decoding phase, an encoding step that includes postprocessing usually comes after the decoding step to mask the influence of the decoding errors. Error or fault concealment usually relies on the production of a redundant data that looks identical to the source. Because of the vast volume of video evidence that is close to itself, it is readily available.

The primary objective of video error concealment systems is to mitigate the effect of failure on picture quality. These schemes use the image's redundancy in the wavelength, spatial, and temporal realms. Under these systems, a portion of the missing data can be retrieved by interpolating the majority of it in any of these realms [2].

Error concealment is a critical feature of every error-tolerant video codec. The effectiveness of an error concealment strategy is heavily contingent on the reliability of the re-synchronization system. In essence, once the re-synchronization mechanism effectively localizes the error, the error concealment problem becomes even more tractable. The current re-synchronization scheme produces very acceptable results for low bit-rate, low delay applications by using a simple concealment strategy, such as copying blocks from the previous image [3].

Data partitioning is an extra error-tolerant mode that improves the decoder's ability to locate errors. The addition of a second re-synchronization marker between the motion and texture data is required by this process. If texture experience is not accessible, this solution employs motion details to mask errors in previously decoded VOPs by motion compensating them [4].

## 2. Motivation and Contribution of the Work

### 2.1. Motivation

One of the most challenging aspects of multimedia communication is recovering damaged or missing images and videos while retaining an acceptable degree of perceived quality. The goal of error concealment is to recover lost data by using frame correlation. Typically, error concealment techniques rely on making a duplicate of the erroneous data that is similar to the original. This is possible because of the high degree of self-similarity of information, such as video. The goal of this study is to provide an error concealing method for frequency-domain video sequences. To conceal defects in movies, they must first be transformed to frames and then into frequency-domain images using one of the many transformations available. It is possible to recover lost or incorrect data from movies by using successive frames.

### 2.2. Contribution of the Work

- This study covers error control methods used in image or video transmission
- Because data are lost during transmission due to connection failure or packet congestion and loss, the goal of this method is to protect data from these errors
- Both the techniques are briefly explained such as error detection coding and error correction coding, and both are types of error control techniques
- Several error control techniques, including re-transmission, forward error correction, error concealment, and error resilience, are also briefly addressed in this study

The remaining sections of this study are organized as follows.  Section 2 discusses related work in the error concealment domain.  Section 3 discusses problem formulation in the error concealment domain.  Section 4 briefly describes tools for error concealment technique.  Section 5 details the planned research work's scope.  Section 6 proposes methodology. Following that, Section 7 introduces the findings of temporal error coverage in a bidirectional manner. Sections 8 and 9 discuss the benefits and drawbacks of error concealment methods, and Section 10 concludes and suggests future work.

## 3. Related Work

Knorr et al. [5] developed and refined an error concealment (EC) method for block losses in stereo pictures. Using a perspective, it identifies feature points surrounding a missing block and compares them to their counterparts in the opposite view. It then constructs a projective mapping from the matched pair of points and employs it to fill in the gaps around the missing block. Clemens et al. [6] modified the maximum smooth recovery technique [7], which was initially suggested for hiding mono pictures and to also include stereo images. They also utilized a projective mapping technique to capitalize on connections between different points of view. Stereo films may hide missing blocks or frames more successfully than mono recordings because they can utilize temporal correlations within each view as well as inter-view correlations. In [8], Guenther et al. presented an error concealing method for block losses in stereoscopic pictures and movies. To minimize the probability of the mistake being discovered, the algorithm substitutes an incorrect block with a motion-compensated or disparity-compensated block, depending on the side match criteria. Pang et al. [9] suggested hiding frame losses in stereo movies using EC techniques. These methods use either motion vector extrapolation or disparity vector cloning techniques. Xiang et al. [10] developed a hybrid EC method for reconstructing a missing block that chooses the best replacement block among motion-compensated blocks, disparity-compensated blocks, or their overlapping blocks. Using these stereo video EC methods [8–10], it is possible to hide erroneous areas in multiview video sequences. Faults in multiview video sequences may be more successfully hidden than defects in stereo video sequences because information from more than two adjacent views may be utilized to hide problems in multiview video sequences.

## 4. Problem Formulation in Error Concealment Domain

**Figure 1:**
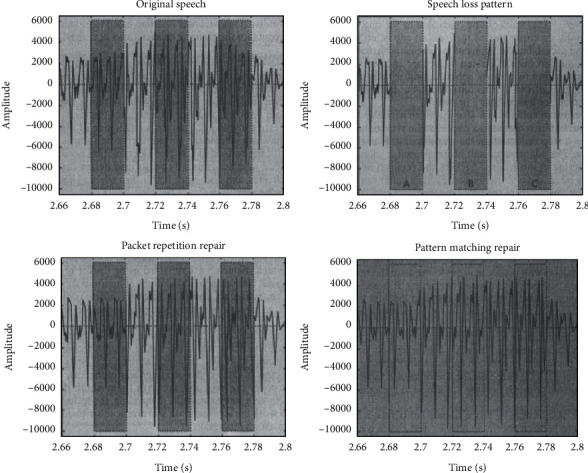
Error concealment [11].

The calculation used to execute the more sophisticated repair techniques rises significantly as compared to the simplified repair alternatives. However, the quality enhancement obtained by these systems is at the best gradual. As a result, using packet replication with fading is suggested as a good balance between achieved efficiency and unnecessary complexity.  Figure 1 depicts a distinction using packet duplication and waveform replacement.

In Figure 1, a distinction using packet duplication and waveform replacement is having following four forms. (a) Techniques for concealing sample errors: original audio signal. (b) Techniques for concealing sample errors: fault pattern. (c) Techniques for concealing sample errors: packet repetition. (d) Techniques for concealing sample errors: one-sided waveform substitution.

Several of these methods may be used for data on only one or both sides of the failure. Many audio and speech coders expect decoder state consistency. When a loss happens, it may be impossible to decipher audio data on both sides of the loss for use in the fix since the decoded audio after the loss may begin in an incorrect condition. Furthermore, two-sided processes have a higher computing overhead and are normally just somewhat better. In certain instances, one-sided repair is adequate [12].

## 5. Tools for Error Concealment

### 5.1. Types of Domains

In general, error concealment research includes three kinds of domains, to name a few: spatial, frequency, and time. This section delves deeply into each topic, starting with the most basic.

#### 5.1.1. Spatial Domain

Assume that the image I is a projection of scene S (which might be a two- or three-dimensional scene). The spatial domain is standard picture space, where a shift in position in I explicitly projects to a change in position in S. Distances in I (in pixels) equate to actual distances in S (in meters, for example).

In spatial concealment, one interpolates directly inside the spatial domain [13], for example, using bi-linear interpolation (if adjacent blocks on all four sides are available), one-dimensional linear interpolation (if only MB above and below are available), or directional interpolation (if only MB above and below are available) (to preserve edges).

#### 5.1.2. Frequency Domain

Assume that image I under scrutiny is the product of a projection from scene S (which may be two-dimensional or three-dimensional). According to [14], the frequency domain is a space in which an image value at image position F reflects the number of how far the intensity values in image I vary from F over a given distance. Changes in image path in the frequency domain correspond to changes in angular frequency (or the rate at which image intensity values change) in image I in the spatial domain.

The spatial frequency domain is attractive in the research field of image processing because

- It may make explicit periodic relationships in the spatial domain
- Some image processing operators are more efficient or indeed only practical when applied in the frequency domain

#### 5.1.3. Time Domain

The concept “time domain” refers to the study of mathematical processes or physical signals in relation to time [15]. In the time domain, the signal or function's significance is known for all real numbers in continuous time or at different distinct instants in discrete time. An oscilloscope is an instrument that is often used to visualize real-world time-domain signals. Temporal concealment employs blocks from other frames. Hybrid algorithms incorporate many methods, including frequency, spatial, and temporal [16].

### 5.2. Types of Errors

Errors that occur in videos can be broadly classified as errors due to following two main reasons:

1. Loss of information in the video
2. Change in spatiotemporal information

Information loss may occur as a result of transmission errors caused by missed blocks. Errors that occur due to mistakes in knowledge distribution lead to image or meaning shift in pixel (e.g., Flicker and noise).

### 5.3. Error Metrics

Because of encoding, transmission, decompression, and other digital effects, video is subject to broad variations. Subjective digital video output can be used for video systems where the video is meant to be watched by people. However, for certain purposes, the same is true. That is because techniques that can calculate how a user feels about perceived picture and video quality would gain more prominence [17].

The most commonly employed metric is the mean square error (MSE), which is the amount of intensity variations of skewed and reference pixels with their corresponding peak signal intensity ratios (PSNR). Mathematically speaking, MSE and PSNR are straightforward to work with and have easily discernible physical definitions. Critics, though, say that they do not relate well to consistency measurements. Therefore, image quality assessment dependent on human judgment is more accurate and a large amount of work has been invested in the creation of qualities that exploit the human visual system (HVS) including blockiness and blurriness (SSIM) [18].

The MSE and the peak signal-to-noise ratio are two separate error metrics that are used to evaluate various image compression techniques (PSNR). MSE denotes the average squared error between the compressed and original files, while PSNR denotes the peak error. Below are the two mathematical formulas:

(1) $$\begin{array}{c} MSE=\frac{1}{MN}\sum_{y=1}^{M}\sum_{x=1}^{N}{\left[\left(x,y\right)-I^{\prime }\left(x,y\right)\right]}^{2}. \end{array}$$

In the preceding equation, *M* and *N* represent the rows and columns of the input images, respectively. The PSNR is then calculated by the block using the following equation:

(2) $$\begin{array}{c} PSNR=20*\;\log\;10\left(\frac{255}{sqrt\left(MSE\right)}\right), \end{array}$$

where *I*(*x*, *y*) is the original image, *I*'(*x*, *y*) is the approximated version (which is actually the decompressed image), and *M* and *N* are the dimensions of the images. A lower value for MSE means a lesser error, and as seen from the inverse relation between the MSE and PSNR, this translates to a high value of PSNR. Logically, a higher value of PSNR is good because it means that the ratio of signal to noise is higher. Here, the ‘signal' is the original image, and the ‘noise' is the error in the frame.

The primary objective of this work is to derive an algorithm for optimum error concealment of online stream video blocks with missing or corrupted packets. This research study suggests a scheme for frequency-domain error concealment that allows good use of all useable knowledge at the decoder. It uses a mathematical model to predict the evolution of transform coefficients from frame to frame and then computes the optimal approximation of the reconstructed coefficient using both current base-layer and previous online stream video-layer results. Additionally, this density-field error concealment (DFEC) scheme naturally allows and complements postprocessing of correctly obtained blocks in order to diagnose and minimize error propagation due to previous losses. The tests obtained significant PSNR improvements.

## 6. Scope for Proposed Work

This study proposes a low-complexity error concealment system for video sequences. The innovation relates to methods and apparatus for concealing errors in transform-domain pictures. Transform coding is a necessary part in many image/video production systems today [19]. Transform coding is based on the assumption that pixels in a picture have a certain degree of similarity with their neighbors. Similarly, parallel pixels in successive frames of a video transmission device show a strong degree of similarity. As a result, these associations may be used to forecast the significance of a pixel based on its neighbors. Thus, a transformation is described as the process by which spatial (correlated) data are transformed into transformed (uncorrelated) coefficients. Clearly, the transition can take advantage of the assumption that an individual pixel's knowledge content is comparatively low, i.e., the visual contribution of a pixel may be predicted to a large degree using its neighbors.

The following steps are implemented in the energy averaging technique:

1. The input video is divided into frames and saved in the database
2. The audio is separated and saved as a .wav file
3. Errors (loss of information and change in information) are introduced in the frames
4. Error frames are saved
5. To correct the error in the frame, the consecutive frame is selected as a reference frame
6. The error frame and the reference frame are converted to frequency domain using transforms
7. The energy matrix of the transformed error frame and reference frame is found
8. All pixels contributing to 90% of the total energy in the error frame is found
9. These pixels are replaced as they are in the corrected frame.
10. For all other pixels, the average pixel value of the error frame energy matrix and the reference frame energy matrix is taken and substituted in the corrected frame
11. Then, the corresponding transform matrix and the inverse transform are found, i.e., the corrected frame is obtained
12. MSE and PSNR of the corrected frame and reference frame are calculated using formulas of (1) and (2)
13. The corrected frame is replaced in place of the error frame and the video is obtained
14. The audio is then added back to the video

Video data can be compressed into a sequence of frames using techniques such as difference coding and are supported by the majority of video encoding specifications, including H.264 [20]. Difference coding compares a picture to a reference frame which codes just the pixels that have shifted in relation to the reference frame. This reduces the amount of pixel values that must be coded and transmitted [21].

## 7. Proposed Methodology

**Figure 2:**
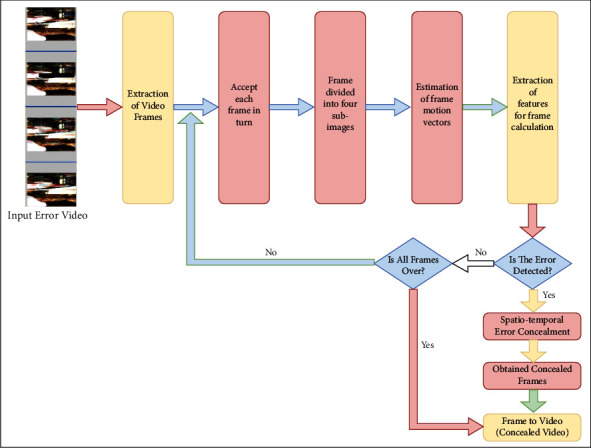
Block diagram of the methodology.

Video error concealment is a technique for hiding errors in video. The demand for error concealment in video applications has recently increased. The Internet, entertainment media, such as television and DVD, video conferencing, and video surveillance all make extensive use of video applications. The goal of this project is to show how to conceal video errors using spatiotemporal image sequence. Errors are detected using frame invariance, and they are repaired using a spatiotemporal image sequence technique. Moment invariance divides the error frame into four subframes and compares them to the previous frames. The proposed algorithm for frame invariance consists of three steps. The first step is to identify a set of possible motion vectors. The second stage involves adaptively calculating the error in the current and reference frames used for feature extraction. The error function is then calculated using frame invariance. The structural similarity index (SSIM) and the peak signal-to-noise ratio determine the video's quality (PSNR). The outcome suggests that the quality of various error videos has improved.  Figure 2 shows a block diagram of the proposed methodology.

The following steps are involved in rearranging pixels:

(i) The input video is divided into frames and saved in database.
(ii) The audio is separated and saved as a .wav file.
(iii) The pixels are rearranged as follows:
  1. The image is divided into pixels
  2. Four new subimages are created
  3. First four pixels of the image are placed in the subimages, respectively
  4. On doing this the four subimages obtained are downsampled versions of the original image
  5. Since most of the information is available in all four subimages, an error in any one of the subimages can now be corrected using any of the other subimages
  6. Once error concealment is applied, the image is again rearranged to obtain the original image

### 7.1. Wavelet Error Concealment

The following steps are involved in wavelet error concealment algorithm.

1. The input video to be corrected is broken down into frames
2. The frames are then subjected to error attacks
3. Two frames, an error frame and a reference frame, are selected from the sequence of frames
4. The frames are converted to frequency domain by applying 3-level wavelet transform
5. Once in the frequency domain, scaled average of both the frames is taken and the averaged value is replaced in the error frame
6. The averaged frame is then converted back to the spatial domain by applying inverse wavelet transform
7. The image obtained on taking the inverse transform is the corrected frame and is almost similar to the reference image
8. The effectiveness of the error concealment algorithm is determined by calculating MSE and PSNR of the corrected frame and the reference frame
9. The corrected frame is then replaced in the sequence of frames and the corrected video is obtained

## 8. Temporal Error Coverage in a Bidirectional Manner

In general, video transmission via the Internet does not ensure service quality. An whole video frame may be lost due to a single packet loss [22]. While most current error concealment techniques can only recover macroblocks, our bidirectional temporal error concealment approach can recover an entire lost frame.

### 8.1. Motion Vector Extrapolation Based on Pixels

Techniques for concealing temporal errors assume that video motion is smooth or continuous. The fundamental approach is to replace the damaged block at the motion-compensated location with the previous frame's content. The drawback of this technique is that it is reliant on motion information, which may not be available in all situations, especially when a whole frame is lost. As a consequence, techniques for estimating lost motion vectors have been thoroughly investigated.

### 8.2. Reverse Estimation

The majority of temporal error concealing methods are entirely dependent on previous frame information. Indeed, since we are unaware of the loss of the current frame until we get the next frame, the information for the next frame is often accessible as well.

### 8.3. Bidirectional Offset

Using the forward and backward methods, we may get two estimations of the present frame. It has been shown that a weighted average of multiple candidate concealment's improves performance [23, 24]. This is a solution that is quite similar to multiple-hypothesis motion compensation (MHMC) [25].

Indeed, we found that simply repeating the motion vectors from the previous frame outperformed all other techniques in stationary or low-motion conditions. A multicovered pixel is one that has several extrapolated MBs covering it. In general, scenes with a lot of motion feature a lot of multicovered pixels.

### 8.4. Advantages of Error Concealment

1. Forward error concealing approaches make a trade-off between efficiency and robustness, necessitating the employment of more sophisticated encoders
2. Postprocessing error concealment techniques do not require additional redundancy and can be applied to any visual system
3. They take advantage of the characteristics of the video signal, and temporal/spatial interpolation is straightforward to apply with noticeable benefits
4. They can be used in conjunction with forward error masking tactics to improve outcomes
5. The cost is the reduction in coding efficiency that these factors induce
6. To develop collaboration between the coder and decoder, interactive error concealment strategies rely on feedback information (which may not be provided in some cases, such as video broadcast systems)
7. They are the ones that have provided the best results thus far because the information is updated at both ends (coder and decoder)
8. Second generation error concealing approaches construct statistical models of the image's various objects and use them to replace the affected area whenever an error occurs
9. Because the model is unique to an object, it can capture the variations in that object more precisely throughout the video series, producing the greatest results of any technique
10. Additionally, the model may be updated online, ensuring that all pertinent data are always available.
11. If the channel being utilized has a very low error rate, the overhead incurred by these approaches is virtually negligible, and their usage may be unnecessary

### 8.5. Disadvantage of Error Concealment

1. The downside is the level of complexity produced throughout the process of developing the numerous statistical models for the items
2. The disadvantage is that they may not be applicable (no feedback channel) and, depending on the system, require more complex encoders and decoders.

## 9. Experimental Results

In this research, proposed algorithm is experimented using a MPEG-5 EVC codec. In the following, we report results related to two parts of the online video frame sequence. The first part ranges from frame 35 to 49 and contains relatively low motion, whereas the second part ranges from frame 180 to 199, and contains fast motion of the camera and the speaker. The sequence has been coded at 300 kbps with 35 fps.

**Figure 3:**
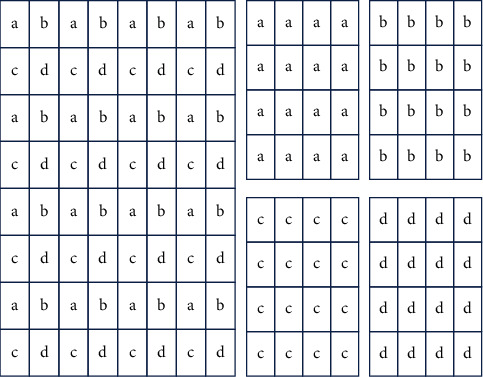
Image divide into four subimages.

**Figure 4:**
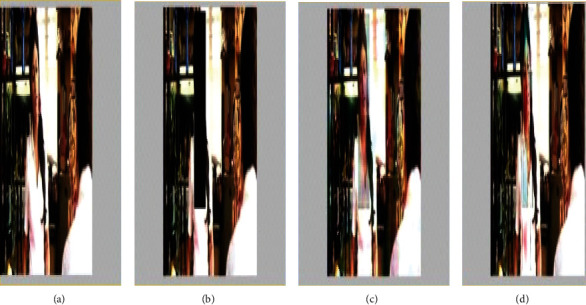
(a) Hartley transform. (b) Haar transform. (c) Hadamard transform. (d) Walsh transform.

**Figure 5:**
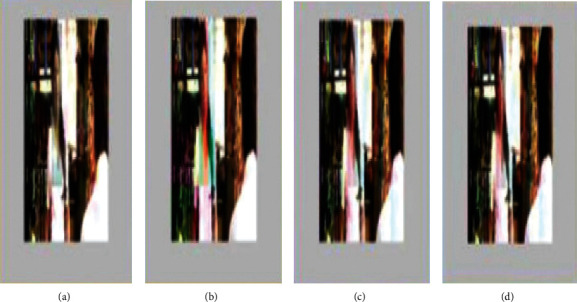
(a) Slant transform. (b) Wavelet. (c) Wavelets' transform. (d) Bewely transform.

**Figure 6:**
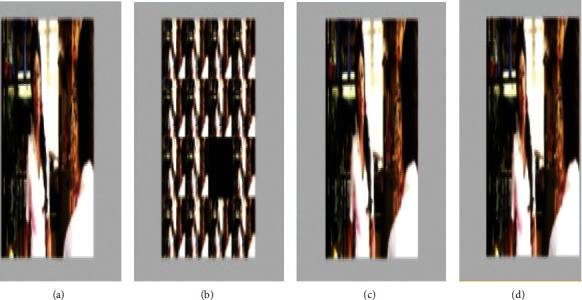
(a) Original frame. (b) Frame with error and corrected image using rearranging. (c) DCT. (d) DST.

**Figure 7:**
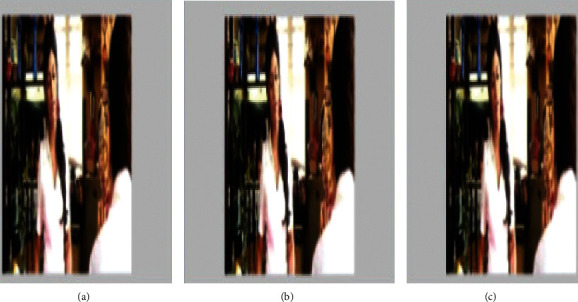
(a) Hartley transform. (b) Haar transform. (c) Hadamard transform.

**Figure 8:**
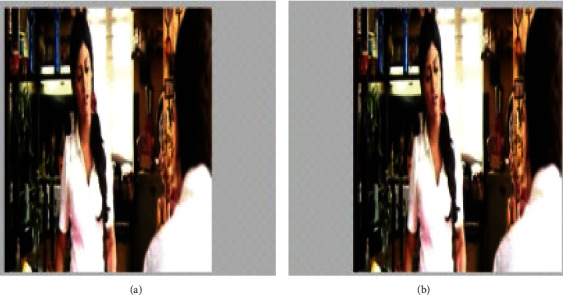
(a) Walsh transform. (b) Slant transform.

Figures 3–8 show the result analysis of the abovementioned algorithms. When the PSNR is compared, the wavelet gives a much better result comparatively.

## 10. Conclusion

Three algorithms for error concealment were built in the proposed project. These algorithms were capable of concealing errors caused by knowledge shift and failure. It is well established that the majority of a frame's energy is contained in its low-frequency components and that the more important in formation is situated in these components. This fact is exploited in this project's energy averaging method, which conceals errors by retaining the pixels that contribute the majority of the frame's energy. After rearranging the pixels, the final image collected includes downsampled copies of the initial file. These downsampled versions may be used to retrieve some missing or incorrect data in the picture, since the majority of information is present in all downsampled versions at various resolutions. The use of wavelet error concealment generated more precise results than the other techniques.

## 11. Future Work

Future work may be done in the video processing extension. It entails using motion vector knowledge to insert the following P-watermark frames in the existing I-frame. Another potential way for this is to find locations in the image/video frame that have little or no motion and embed the watermark there. This may be advantageous, since audiences often concentrate on locations with a lot of motion. Another potential future path is to develop a rigorous watermarking methodology for this program. Due to the simplicity of which spread spectrum watermarking can be implemented in the DCT domain, it was used here as a proof of concept. However, due to its strong spreading advantage and increased entropy, it is not optimal for this use. Watermarking using quantization can be an option. A robust watermarking methodology suitable for error control applications is also lacking.

## Data Availability

The data that support the findings of this study are available from the corresponding author upon request.
